# Peripheral Ion Channel Genes Screening in Painful Small Fiber Neuropathy

**DOI:** 10.3390/ijms232214095

**Published:** 2022-11-15

**Authors:** Milena Ślęczkowska, Rowida Almomani, Margherita Marchi, Erika Salvi, Bianca T A de Greef, Maurice Sopacua, Janneke G J Hoeijmakers, Patrick Lindsey, Stephen G Waxman, Giuseppe Lauria, Catharina G Faber, Hubert J M Smeets, Monique M Gerrits

**Affiliations:** 1 Department of Toxicogenomics, Maastricht University, 6229 ER Maastricht, The Netherlands; 2 Department of Neurology, School of Mental Health and Neuroscience, Maastricht University Medical Centre+, 6229 ER Maastricht, The Netherlands; 3 Department of Medical Laboratory Sciences, Jordan University of Science and Technology, Irbid 22110, Jordan; 4 Neuroalgology Unit, IRCCS Foundation “Carlo Besta” Neurological Institute, 20133 Milan, Italy; 5 Department of Neurology, Yale University School of Medicine, New Haven, CT 06510, USA; 6 Center for Neuroscience and Regeneration Research, Yale University School of Medicine, New Haven, CT 06510, USA; 7 Department of Clinical Genetics, Maastricht University Medical Centre+, 6229 HX Maastricht, The Netherlands

**Keywords:** idiopathic small fiber neuropathy, ion channel, MIPs-NGS, neuropathic pain, peripheral neuropathy

## Abstract

Neuropathic pain is a characteristic feature of small fiber neuropathy (SFN), which in 18% of the cases is caused by genetic variants in voltage-gated sodium ion channels. In this study, we assessed the role of fifteen other ion channels in neuropathic pain. Patients with SFN (n = 414) were analyzed for *ANO1*, *ANO3*, *HCN1*, *KCNA2*, *KCNA4*, *KCNK18*, *KCNN1*, *KCNQ3*, *KCNQ5*, *KCNS1*, *TRPA1*, *TRPM8*, *TRPV1*, *TRPV3* and *TRPV4* variants by single-molecule molecular inversion probes–next-generation sequencing. These patients did not have genetic variants in *SCN3A*, *SCN7A-SCN11A* and *SCN1B-SCN4B*. In twenty patients (20/414, 4.8%), a potentially pathogenic heterozygous variant was identified in an ion-channel gene (ICG). Variants were present in seven genes, for two patients (0.5%) in *ANO3*, one (0.2%) in *KCNK18*, two (0.5%) in *KCNQ3*, seven (1.7%) in *TRPA1*, three (0.7%) in *TRPM8*, three (0.7%) in *TRPV1* and two (0.5%) in *TRPV3*. Variants in the TRP genes were the most frequent (n = 15, 3.6%), partly in patients with high mean maximal pain scores VAS = 9.65 ± 0.7 (n = 4). Patients with ICG variants reported more severe pain compared to patients without such variants (VAS = 9.36 ± 0.72 vs. VAS = 7.47 ± 2.37). This cohort study identified ICG variants in neuropathic pain in SFN, complementing previous findings of ICG variants in diabetic neuropathy. These data show that ICG variants are central in neuropathic pain of different etiologies and provides promising gene candidates for future research.

## 1. Introduction

Neuropathic pain (NeuP) is defined as a pain condition usually caused by progressive nerve disease [1]. NeuP symptoms are often described as a shooting or burning pain accompanied by allodynia, hyperalgesia, sensory dysfunction and autonomic complaints [2]. Chronic pain is common in peripheral neuropathy, including diabetic neuropathy (DN) and small fiber neuropathy (SFN), where Aδ-fibres and C-fibres are affected [3,4]. Patients suffering from neuropathic pain report major negative impact on quality of life [5]. Unfortunately, the currently available treatment has a moderate effect and often does not bring the expected pain relief [2,6]. Several conditions such as diabetes mellitus, autoimmune disorders, viral infections, inflammatory disorders and chemotherapy have been linked to NeuP, but the pathophysiology is largely unresolved [2]. An increasing number of reports highlight a role for genetic factors involved in pain development [1,7,8]; still, in more than 80% of the cases, a possible genetic factor is unknown [9].

In the last two decades, alterations of voltage-gated sodium ion channels (VGSCs) have been reported to be caused by genetic mutations in the underlying genes [10,11,12,13]. VGSCs are transmembrane polypeptides responsible for the generation and conduction of action potentials in excitable cells [14]. Gain-of-function (GOF) variants of *SCN9A*, *SCN10A* and *SCN11A* have been reported in several pain-related diseases, including SFN [10,11,12,14,15], adding up to 12% in patients with pure SFN [3]. Screening of all VGSCs genes, including *SCN3A*, *SCN7A-SCN11A* and *SCN1B-SCN4B*, increased the number of patients with NeuP with an identified (potential) underlying cause to 18.1% [9].

In the literature, other ion channels’ genes (ICGs) have also been reported in pain modulation, mainly transient receptor potential (TRP) cation channels [16], potassium voltage-gated (Kv) channels [17], hyperpolarization-activated and cyclic nucleotide-gated channels (HCN) [18] and Ca^2+^-activated Cl- channels, also known as anoctamins (ANO) [19]. TRP channels function as thermal, chemical and mechanical sensors [16]. Kv are a group of potassium channels involved in the modulation of sensory neuron excitability and pain processing [17]. HCN channels exhibit wide expression in peripheral nerves, and their impaired functioning has been linked to neuropathic pain [18]. The most studied member of the ANO family, ANO1, has been found to interact with TRPV1, leading to increased pain in sensory neurons [20]. Moreover, ANO3 modulates nociception in the dorsal root ganglion (DRG) via enhancement of the sodium-activated potassium channel Slack activity [21].

In a previous study, we investigated the role of variants in 15 ICG in different patient cohorts with painful and painless DN. GOF and LOF variants were present in both groups, suggesting that ICG variants contribute to NeuP [22]. Therefore, in this study, we extended the analysis of these ICG to a different patient cohort of neuropathic pain, SFN. We focused specifically on patients with SFN without a defined underling genetic cause, as analysis of the *SCN3A*, *SCN7A-SCN11A* and *SCN1B-SCN4B* genes was negative. We performed single-molecule molecular inversion probes–next-generation sequencing (smMIPs-NGS) to analyze exons and exon–intron junctions and classified the identified variants.

## 2. Results

### 2.1. smMIPs-NGS of Patients with SFN

**Figure 1:**
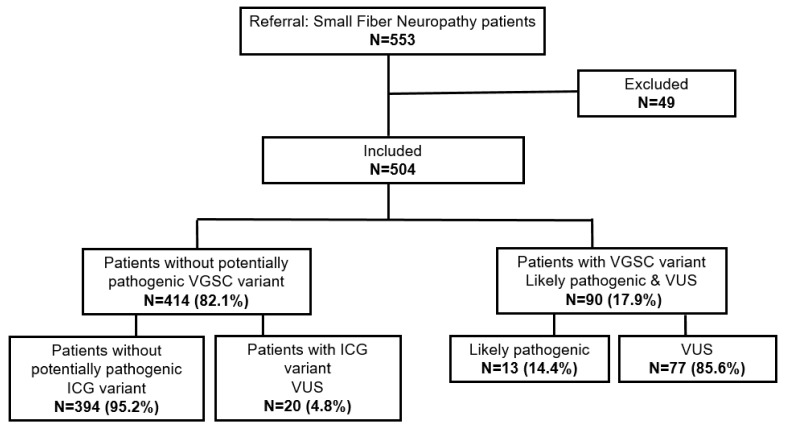
SFN patients analyzed using smMIP-NGS for (potentially) pathogenic ICG and VGSC variants. ICG, ion channel gene; VGSC, voltage-gated sodium ion channel; VUS, variant with uncertain clinical significance.

The performance of smMIPs-NGS, capture efficiency and coverage of targeted exons and exon-flanking sequences (±20 bps) for a 15-ICG panel were assessed as described previously [22]. The average coverage of these regions (>30x/bp) was 93%. The targeted sequences of *ANO3* (2946 bp), *KCNA2* (1500 bp), *KCNA4* (1962 bp), *KCNK18* (1155 bp), *KCNQ3* (2619 bp), *KCNQ5* (2856 bp), *TRPA1* (3360 bp), *TRPM8* (3315 bp), *TRPV1* (2520 bp) and *TRPV4* (2616 bp) had an average coverage >90%, and for five targeted gene areas (*ANO1* (2961 bp), *HCN1* (2673 bp), *KCNN1* (1632 bp), *KCNS1* (1581 bp), *TRPV3* (2376 bp)) the average coverage was at least 84%. Four exons (exon 1, exon 12 and exon 20 of *ANO1*, and exon 3 of *TRPV1*) had poor coverage (<20x/bp) or were completely missing. The calculated capture efficiency of each probe and coverage of on-target regions was reproducible per region, per run and between samples. A total of 49 out of 553 samples were excluded due to low quality of DNA, insufficient number of reads or incomplete clinical information (Figure 1).

### 2.2. Genetic Variants Identified in Ion Channel Genes

Among 414 patients negative for *SCN3A*, *SCN7A-SCN11A* and *SCN1B-SCN4B* screening, 19 different potentially pathogenic variants were identified in ICGs. All detected variants were classified as VUS and localized in seven ICG genes: *ANO3*, *KCNK18*, *KCNQ3*, *TRPA1*, *TRPM8*, *TRPV1* and *TRPV3*. In total, two missense variants were detected in *ANO3*, one frameshift variant leading to a shorter protein in *KCNK18*, two missense in *KCNQ3*, four missense and two nonsense variants in *TRPA1*, three missense in *TRPM8*, three missense in *TRPV1*, one splice variant in the donor site of intron 9 and one missense in *TRPV3* (Table 1). All of them were heterozygous. The detected nonsense/frameshift variants were predicted either to cause a shortening of the protein length due to a premature stop codon or, most likely, cause nonsense-mediated decay (NMD) of the mRNA, while the splice site variant in the donor site of intron 9 (*TRPV3* c.1242+1G>A) was predicted to alter the protein sequence via alternative splicing.

Although the best predictable functional effect will be lack of the protein due to NMD or truncated, nonfunctional polypeptides (*KCNK18* c.1107del, *TRPA1* c.1177C>T and c.1954C>T), the substitution in conserved functional areas of the protein may affect proper functioning. Three variants (*TRPV1* c.1348A>G and c.1735C>T and *TRPV3* c.2006T>C) were found in transmembrane domains. One variant (*ANO3* c.2656A>T) was detected in a linker between transmembrane domains, three variants were located in ankyrin repeats (ANK) (*TRPA1* c.932C>A and c.980A>G and *TRPV1* c.914T>G), three in the N-terminus (*TRPM8* c.665A>G and c.1102C>T), four in the C-terminus (*ANO3* c.3100G>C, *KCNQ3* c.1885G>A and c.1706A>G, *TRPA1* c.3136A>G and *TRPM8* c.2945C>T) and one in the inositol-phosphate binding site of TRPA1 (c.3136A>G).

Each ICG variant was detected in one patient, with the exception of *TRPA1* c.3136A>G, which was present in two siblings affected by SFN (Table 1). Most of the reported ICG variants were novel (n = 16/19, 84%). The *TRPA1* c.1954C>T variant has been seen before in a German painful-diabetic-peripheral-neuropathy patient [22]. The majority of the identified (n = 14/19, 73.7%) variants were located in TRP genes. No potential causing variant was identified in eight genes from our gene panel: *ANO1*, *HCN1*, *KCNA2*, *KCNA4*, *KCNQ5*, *KCNN1*, *KCNS1* and *TRPV4*. None of the patients with an identified VGSC variant had an additional variant in one of the other ICGs (Figure 1).

### 2.3. Patients with SFN with an ICG Variant Compared to Patients without ICG/VGSC Variant

We did not observe statistically significant differences between patients with a potentially pathogenic ICG variant compared to patients without such a variant (no ICG/VGSC variant) in relation to mean age of onset of neuropathy (52.2 ± 13.3 vs. 54.3 ± 13.9 years old, *p* = 0.786), neuropathy duration (n = 12, 4.4 ± 5.0 years vs. n = 277, 8.1 ± 9.3, *p* = 0.183), positive family history (n = 5, 38.5% vs. n = 50, 22.2%, *p* = 0.088), abnormal TTT (n = 9, 100% vs. n = 280, 91.8%, *p* = 1) and abnormal skin biopsy (n = 14, 77.8% vs. n = 129, 42%, *p* = 0.0056) (Table 2). Detailed clinical characteristics of patients with SFN and an ICG variant are available in the Supplementary Materials.

During the clinical assessment, multiple questionnaires, including the SFN Symptom Inventory Questionnaire (SFN-SIQ) [23] and the Visual Analogue Scale, were given to the patients (Table 3). All analyzed patients that completed the questionnaire reported severe pain; however, individuals carrying a potentially pathogenic ICG variant (n = 7) had higher maximal pain scores VAS = 9.36 ± 0.72 compared to those in the group without such an ICG/VGSC variant (n = 204, VAS = 7.47 ± 2.37 (Table 3)). Each individual with ICG reported maximal pain above the mean maximal pain calculated for patients without an ICG/VGSC variant. The patient with an *ANO3* variant reported maximal pain (VAS = 9.7), the patient with *KCNQ3* c.1885G>A variant reported VAS = 8.7, and the patient with *KCNQ3* c.1706A>G reported VAS = 8.5. Interestingly, three out of four *TRPA1*-positive patients evaluated maximal pain as VAS = 10, which is the highest possible value in the scale. Only one patient carrying the *TRPA1* variant, specifically c.2065A>G, reported VAS = 8.6. Therefore, the maximal pain scores in the group of patients having variants in the same gene were higher than those in the subgroup without an ICG/VGSC variant (Table 3).

Several features, including SFN-specific symptoms like a changed sweating pattern, diarrhea, constipation, micturition problems, dry eyes, dry mouth, orthostatic dizziness, palpitations, hot flashes, sensitive leg skin, burning feet, sheet intolerance legs and restless legs were evaluated for the SFN patients (Table 4 and Table 5). All patients with an ICG variant for which these data were available (n = 6) reported often or always burning feet and the restless legs symptom. Next to that, the most commonly mentioned complaint among patients with ICG variants was sheet intolerance on legs, which presented often or always (n = 5/6, 83.3%) and sometimes (n = 1/6, 16.7%) (Table 6). However, burning feet, sheet intolerance and restless legs were also the most commonly mentioned features in patients without an ICG/VGSC variant. Burning feet presented always and often (n = 213, 79.1%), restless legs presented always and often (n = 156, 57.4%) and sheet intolerance presented always and often (n = 126, 46.3% (Table 7)). Statistical significance was not reached for any of investigated features.

## 3. Discussion

### 3.1. Summary of ICG Screening in Patients with Small Fiber Neuropathy

In 414 patients with no (potentially) pathogenic variants in the *SCN3A*, *SCN7A-SCN11A* and *SCN1B-SCN4B* genes, twenty patients (4.8%) had one potentially pathogenic heterozygous variant in ICG. The detected variants were located in seven genes and were present in two patients (0.5%) in *ANO3*, one (0.2%) in *KCNK18*, two (0.5%) in *KCNQ3*, seven (1.7%) in *TRPA1*, 3 (0.7%) in *TRPM8*, three (0.7%) in *TRPV1* and two (0.5%) in *TRPV3*. Most of the detected variants were novel; however, three variants have been reported before. The *ANO3* c.3100G>C variant is present in the Human Gene Mutation Database (HGMD) and described as likely disease-causing with questionable pathogenicity in primary torsion dystonia [24]. Two *KCNQ3* variants have been reported in ClinVar; c.1885G>A was reported two times, once as a VUS in benign familial neonatal seizures and as once as likely benign without linking to a specific phenotype, while the c.1706A>G variant was reported as a VUS, and the phenotype was not provided. Our patients did not have complaints typical for dystonia and familial neonatal seizures.

In our study population, each ICG variant was detected only once, with exception of the *TRPA1* c.3136A>G variant present in a brother and sister with SFN, which was considered as one independent finding. None of the identified ICG variants have been reported before in neuropathic pain, except for the *TRPA1* c.1954C>T variant, which we published recently, present in a 73-year-old male diagnosed with painful DN [22].

The frequency of potentially pathogenic ICG variants in our population (4.8%) was slightly lower than the frequency in patients with painful DN (5.4%) [22]. The most frequent variants in the SFN cohort were located in TRP genes, in total in fifteen patients (3.6%), which is consistent with data obtained for painful DN, where seven individuals (3.3%) had identified VUS TRP variants, three (1.4%) in *TRPA1*, three (1.4%) in *TRPM8*, one (0.5%) in *TRPV4*. Different heterozygous missense VUS were also present in three (1.4%) painful DN patients in *ANO3* and two different *KCNK18* variants leading to a premature stop codon in two (0.9%) individuals with painful DN [22]. Therefore, our data suggest that although the same genes may be involved, different variants underlay SFN and painful DN. No potentially pathogenic variants were detected in eight genes: *ANO1*, *HCN1*, *KCNA2*, *KCNA4*, *KCNQ5*, *KCNN1*, *KCNS1* and *TRPV4*. Five of them, *KCNA2*, *KCNA4*, *KCNQ5*, *KCNN1* and *KCNS1*, were also negative for screening in a diabetic neuropathy population [22]. Interestingly in an SFN cohort, variants have been detected in three genes: *KCNQ3*, *TRPV1* and *TPRV3*, which did not appear in painful DN [22]. Therefore, although it seems that ICG variants can play a role in DN and SFN, it cannot be concluded due to the limited cohort sizes that specific ICG or ICG genes are responsible for either clinical manifestation. The list of genes, however, is a promising candidate for future research.

### 3.2. VUS Meaning in Context of Protein Function

All detected variants were classified as VUS, which reflects the limitations of bioinformatics analysis of novel variants in absence of functional data or segregation in the family [25,26]. In this study, we only relied on in silico prediction of pathogenicity, therefore, it is difficult to draw a definite conclusion about variant causality, which demands further validation. However, it is likely that a number of these VUS will change to (possibly) pathogenic, as they clearly have a strong impact on the protein. The nonsense/frameshift/splice site variants cause altered protein length or protein absence due to nonsense-mediated decay (NMD). Moreover, substitutions are present in conserved, functionally relevant regions of the protein, most likely affecting protein function, like channel opening and heat sensitivity (variants in the transmembrane domain) [27], channel gating and desensitization (variants in the linker between transmembrane domains) [28], protein–protein interactions (variation in ANK) [29,30], protein stability and folding (variants in N- and C-terminus) [31] and binding sites, such as the inositholphospathe binding site in the case of *TRPA1* c.3136A>G, leading to channel inactivation [32].

### 3.3. ICG Variants in Relation to Patients’ Clinical Manifestations

#### 3.3.1. Anoctamin 3

Anoctamin 3 is a member of the Ca^2+^-activated chloride channel family involved in pain processing and thermoregulation [33]. *ANO3* indirectly inhibits pain signaling via enhancing sodium-activated potassium (SLACK) channels in DRG [21]. Since *ANO3* knockout results in a decreased pain threshold in tested rats, GOF variants are expected in pain-related phenotypes [21]. Consistent with that, two heterozygous missense variants with predicted GOF were detected in individuals affected with painful DN [22]. In our study cohort of SFN patients, two heterozygous missense variants (*ANO3* c.2656A>T and c.3100G>C) have been identified in two unrelated subjects. Both of them reported burning pain, allodynia and sheet intolerance. TTT was abnormal in both cases; moreover, a female patient identified cold temperature as a pain-provoking factor. Altogether, it may link the patients’ clinical manifestation with disrupted thermoregulation.

#### 3.3.2. Potassium Channels

Potassium channels are important in regulation of nociceptor excitability in sensory neurons [34]. A heterozygous deletion of one nt in *KCNK18* c.1107del leading to a frameshift and a shortened C-terminus has been identified in an SFN patient. Although missense and frameshift mutations with a premature stop codon in *KCNK18* are linked mainly to migraine, some studies indicate that downregulation of *KCNK18* contributes to neuropathic pain [35,36]. Two patients from our cohort harbored two different heterozygous missense variants in *KCNQ3* c.1885G>A and c.1706A>G. Interestingly, both of them had a similar phenotype with numb sensation and burning pain in the feet. In both cases, pain complaints were getting worse at rest. Additionally, both patients had disturbed warm and cold sensation in their feet. However, one patient reported that cold increased and warmth reduced pain symptoms, while the other patient did not indicate a temperature influence on pain. GOF mutations in *KCNQ3* are recognized as contributors to pain resilience [37], therefore, a loss-of-function (LOF) effect of detected variants would be expected in our patients with SFN and neuropathic pain.

#### 3.3.3. TRP Channels

Variants located in the TRP genes constitute the largest group of variants identified in our study cohort. TRP channels constitute a large, divergent gene family with a well-documented role in mediating nociceptive behaviors in response to thermal, chemical and osmotic stimuli [38]. In total, six variants were detected in *TRPA1* (two nonsense and four missense), three missense variants in *TRPM8*, three missense in *TRPV1* and two variants in *TRPV3* (one missense and one splice variant). TRPA1 channel activity has been functionally linked to pain hypersensitivity, cold hyperalgesia and mechanical nociception in inflammatory and neuropathic pain models [39,40,41]. The GOF mutation N855S in *TRPA1* has been associated with familial episodic pain syndrome. The TRPA1 R919* variant is segregating in a family with the cram-fasciculation syndrome [42,43]. Interestingly, the R919* GOF effect was suggested based on clinical improvement with carbamazepine; however, it was not demonstrated whether the truncated TRPA1 protein was expressed at all [43]. In our study, one patient heterozygous for the missense VUS c.980A>G had complaints of numb sensation and painful cramps in the fingers, lower legs and feet, worsening after exercise. Several VUS leading to premature stop codons or nonsense-mediated RNA-decay variants have been reported in painful DN [22], and with the presence of two nonsense variants in our patient cohort (c.1177C>T and c.1954C>T in painful DN), a LOF mechanism would in our opinion be more likely. The clinical manifestation in the patients with *TRPA1* VUS was diverse in our cohort; however, the most common complaints were numb sensation, tingling and burning pain. Four out of seven patients reported pain increase after exercise, and all the patients with performed TTT had abnormal TTT values.

Three different heterozygous VUS have been detected in patients with disturbed thermal sensation (abnormal TTT or QST) in *TRPM8*. Additionally, the patient with c.665A>G VUS reported increased pain due to warm temperature. As *TRPM8* is a modulator of cold sensation and a potential target for neuropathic pain treatment [38], those variants remain interesting candidates for functional validation and determine a GOF and LOF effect.

*TRPV1* and *TRPV3* are heat-activated proteins involved in thermosensation and pain perception [38]. In addition to that, *TRPV3* activation plays a role in enhanced itch sensation [44]. In our study, two patents had a *TRPV3* variant (c.20006T>C combined with itch episodes and a splice-site variant c.1242+1G>A *r.spl?*), and three patients were heterozygous for a missense variant in *TRPV1* (c.914T>G or c.1348A>G or c.1735C>T). Two of them reported increased pain complaints during rest, which might interfere with night rest. Interestingly, no pain change was reported in response of warm/cold temperature. The literature indicates that upregulation of *TRPV1* increases amplification of pain signals [38]. Consistently with that, the GOF Q85R mutation of *TRPV1* has been linked to neuropathic pain [45]. On the other hand, *TRPV1* missense variants localized in transmembrane domains have been reported in diabetic neuropathy patients without pain [22], which highlights the need of functional variant validation before drawing a definite conclusion.

### 3.4. Patients with an ICG Variant Had More Severe Clinical Manifestations and Higher Pain Scores

Due to the small number of patients with an ICG variant, it is difficult to draw a definite conclusion whether these patients had more severe clinical manifestations in relation to basic clinical features or SFN-SIQ when compared to those of patients without an ICG/VGSC variant. It seems that patients with an ICG variant tended to have more frequent abnormal skin biopsies and a shorter duration of neuropathy; however, statistical significance was not reached for any of these features. There is evidence implicating enhanced Na^+^ influx produced by gain-of-function Nav1.7 mutations as a trigger for calcium-importing reverse Na^+^/Ca^2+^ exchange that can contribute to axonal degeneration [46]; further work will be needed to determine whether ionic imbalances due to any of the variants found in this study are injurious to axons. Nevertheless, patients with an ICG variant reported higher maximal pain VAS = 9.36 ± 0.72 than patients without an ICG/VGSC variant VAS = 7.47 ± 2.37 did. All of the patients with an ICG variant had severe mean maximal pain VAS ≥ 8.5, always higher than the mean calculated for patients without an ICG/VGSC variant. Interestingly, patients with *TRPA1* reported very severe pain (mean max pain VAS = 9.65 ± 0.7), and three of them gave VAS = 10, which is the highest possible score. These data are consistent with a previous study showing that mean maximal pain was higher for painful DN patients with an ICG variant vs. painful DN without an ICG variant [22].

## 4. Material and Methods

### 4.1. Study Population

From June 2014 to September 2016, 553 patients diagnosed with SFN were recruited at Maastricht University Medical Center+ (MUMC+), Maastricht, The Netherlands and at Fondazione I.R.C.C.S. Istituto Neurologico Carlo Besta (FINCB), Milan, Italy for this study. MUMC+ is a tertiary referral center for SFN in the Netherlands, and FINCB is a national referral center for several neurological conditions, including SFN. Medical history and clinical data were collected and recorded as described previously [12]. The diagnosis of SFN was confirmed based on clinical symptoms of SFN combined with reduced intraepidermal nerve fiber density (IEND) in skin biopsy without signs of large fiber involvement and/or abnormal temperature threshold testing (TTT) and/or nerve conduction study (NCS) [47]. The assessment of neuropathic pain was performed using the visual analog scale (VAS), where the scores are recorded by marking a value on a 10 cm line in which 0 represents “no pain” and 10 is “the worst pain that you can imagine”, and the 11-point Numerical Rating Scale (PI-NRS) ranging from 0 to 10, where 0 means “no pain” and 10 refers to “the worst pain imaginable” [48,49]. Only patients with diagnosed SFN and neuropathic pain with VAS > 3 were included. In this study, we focused on patients negative for *SCN3A*, *SCN7A-SCN11A* and *SCN1B-SCN4B* variant screening. Most of the included patients (n = 348, 69%) were Dutch, while 156 (31%) patients were from Italy. The mean age of recruited SFN patients was 54.1 years (SD 14.2 years), with the mean age of complaints onset of 42.2 years (SD 13.6 years). More females than males were present in the study population. There were 59.5% (n = 300) of females and 40.5% (n = 204) of males. In both populations, females were over-represented, with 59.8% (n = 208) females in the Dutch cohort and 59% (n = 92) females in the Italian cohort, respectively. Twenty-three percent of the patients (n = 65) reported positive cases of neuropathy in the family, while negative family history was registered for 77% (n = 217). The data for familial cases of neuropathy was incomplete for 222 patients. More than one-third of the recruited patients had an abnormal skin biopsy (n = 120, 36.5%).

### 4.2. DNA Extraction and Storage

Genomic DNA was from blood samples using a NucleoSpin8 Blood Isolation kit (Macherey-Nagel, Düren, Germany) or a QIAamp DNA Blood Maxi Kit, Puregene^®^ Blood Core Kit (Qiagen, Hilden, Germany). DNA extraction was performed according to the manufacturers’ instructions and stored in −20 °C.

### 4.3. smMIPs-NGS of Peripheral Ion Channels and Data Analysis

The smMIP-NGS of exonic regions with ±20 bps exon-flanking sequences of the 15 ion channel genes (Table 8) was performed according to a previously established protocol [50]. The detailed description of the technique, smMIPs characteristics, number of probes per targeted coding region per gene and smMIPs-NGS pipeline used for data analysis have been described before [22].

### 4.4. Variant Classification

Exonic and ±20 bp exon-flanking intron variants with >20x coverage and an alternative variant call of >30% were included for the analysis. The SNPs with high frequency, defined as dbSNP > 5%, ExAC > 5% and >1% in our Dutch in-house database of 12,244 exomes, were excluded from the analysis. All variants were examined individually, including BAM file visualization and variant interpretation performed in Alamut Visual software (Interactive Biosoftware, Rouen, France). Variants were classified according to the practice guidelines of the Association for Clinical Genetic Science (ACGS) [51]. 

### 4.5. Statistical Analysis

The analysis of continuous variables was performed using the independent Student’s *t*-test. Categorical variables were analyzed using the chi-square test or Fisher’s exact test in the case of small counts <5. Statistical significance of <0.05 was applied.

## 5. Conclusions

In a well-characterized cohort of patients with SFN, we identified twenty patients (4.8%) carrying a potentially pathogenic variant in ICG. Patients with an ICG variant might have more severe pain than patients without a (potentially) pathogenic ICG/VGSC variant. This complements previous results of the analysis of the same ICG in a large cohort of painful and painless DN. Moreover, it extends the list of potential pain-related genes, reporting variants in *KCNQ3*, *TRPV1* and *TPRV3* that were not present in painful DN. Apparently, ICGs are fundamental for both SFN and DN, although the numbers are too small to unambiguously identify gene-or variant-specific manifestations. Although all of the variants were classified as VUS and the consequences of the variation cannot be certainly predicted in silico, they are still promising risk factors for neuropathic pain as well as promising gene candidates for future research.

## Figures and Tables

**Table 1:** Potentially pathogenic variants of ion channel genes identified in patients with SFN (n = 504).

Gene	c.Position ^&^	p.Position	Number of Patients	Classification According to Richards et.al 2015	Location	MAF gnomAD (%)
*ANO3*	c.2656A>T	p.(Ile886Phe)	1	VUS	Linker between transmembrane domain VII and VIII	0
	c.3100G>C	p.(Gly1034Arg)	1	VUS	C-terminus	0.011
*KCNK18*	c.1107del	p.(Met370Cysfs*?)	1	VUS	Frame shift starting at codon Met370	0.0016
*KCNQ3*	c.1885G>A	p.(Val629Ile)	1	VUS	C-terminus	0.052
	c.1706A>G	p.(Asp569Gly)	1	VUS	C-terminus	0.0018
*TRPA1*	c.932C>A	p.(Thr311Asn)	1	VUS	Ankyrin repeat VIII-containing domain	0.044
	c.980A>G	p.(Tyr327Cys)	1	VUS	Ankyrin repeat VIII-containing domain	0
	c.1177C>T	p.(Arg393*)	1	VUS	Stop codon in Ankyrin repeat X–containing domain	0.018
	c.1954C>T	p.(Arg652*)	1	VUS	Cytoplasmic domain between ANK repeats and transmembrane domain I	0.015
	c.2065A>G	p.(Met689Val)	1	VUS	Cytoplasmic domain between ANK repeats and transmembrane domain I	0.0068
	c.3136A>G	p.(Lys1046Glu)	2 *	VUS	inositol-phosphate binding site in C-terminus	0.0008
*TRPM8*	c.665A>G	p.(Asn222Ser)	1	VUS	N-terminus	0.0004
	c.1102C>T	p.(Arg368Trp)	1	VUS	N-terminus	0.0016
	c.2945C>T	p.(Thr982Met)	1	VUS	C-terminus	0.005
*TRPV1*	c.914T>G	p.(Phe305Cys)	1	VUS	Ankyrin repeat V-containing domain	0.00054
	c.1348A>G	p.(Thr450Ala)	1	VUS	Transmembrane domain I	0
	c.1735C>T	p.(Arg579Cys)	1	VUS	Transmembrane domain V	0.0017
*TRPV3*	c.1242+1G>A	p.? ^^^	1	VUS	Donor splice site of intron 9	0.0029
	c.2006T>C	p.(Leu669Pro)	1	VUS	Transmembrane domain VI	0.0019

c. position, location cDNA; p. position, location in protein; MAF gnomAD, minor allele frequency; VUS, variant with uncertain clinical significance. ^&^ Variants detected were annotated according to the guidelines of the Human Genome Variation Society using reference sequence GRCh37 and transcript numbers, NM_001313726.1 (*ANO3*); NM_181840.1 (*KCNK18*), NM_004519.3 (*KCNQ3*), NM_007332.2 (*TRPA1*), NM_024080.4 (*TRPM8*), NM_080706.3 (*TRPV1*)*,* NM_001258205.1 *(TRPV3*). All detected variants are heterozygous. * Variant detected two times in siblings, ^^^ changed protein length due to splicing event (loss of donor splice site of intron 9).

**Table 2:** Comparison of clinical features of patients with small fiber neuropathy and ICG variant vs. no ICG/VGSC variant.

	Patients with SFN and ICG Variant N = 20	Patients with SFN without ICG/VGSC Variant N = 394
Mean age at recruitment [years ± SD]	52.2 (±13.3)	54.2 (±13.9)
Females (n, %)	11 (55.0)	240 (±60.9)
Males (n, %)	9 (45.0)	154 (±39.1)
Mean age of onset neuropathy [years ± SD]	46.2 (±12.2)	47.1 (±13.4)
Duration of neuropathy [years ± SD]	4.4 (±5.0)	8.1 (±9.3)
Positive family history for neuropathy (n, %)	5 (38.5)	50 (22.2)
Negative family history for neuropathy (n, %)	8 (61.5)	175 (77.8)
Normal skin biopsy (n, %)	4 (22.2)	178 (58)
Abnormal skin biopsy (n, %)	14 (77.8)	129 (42)
Normal TTT (n, %)	0 (0)	25 (8.2)
Abnormal TTT (n, %)	9 (100)	280 (91.8)

ICG, ion channel gene; VGSC, voltage-gated sodium ion channel; SD, standard variation. Patients with incomplete data not included in the table. Differences between patients with SFN and ICG variant vs. patients with SFN without ICG and VGSC variant were not statistically significant.

**Table 3:** Mean pain scores in patients with small fiber neuropathy.

	Patient with ANO3 Variant (n = 1)	Patients with KCNQ3 Variant (n = 2)	Patient with TRPA1 Variant (n = 4)	Patients with ICG Variant (n = 7)	Patients without ICG Variant and without VGSC Variant (n = 204)
Maximal pain [VAS] [±SD]	9.7	8.6 (±0.14)	9.65 (±0.7)	9.36 (±0.72)	7.47 (±2.37)

ICG, ion channel gene; VGSC, voltage-gated sodium ion channel; SD, standard variation. Pain evaluated using the visual analog scale (VAS), where 0 represents “no pain” and 10 is “the worst pain that you can imagine”. Patients with incomplete data not included in the table.

**Table 4:** Clinical features of SFN patients carrying an ICG variant.

Gene	Variant	Gender	Onset Complaints	NCS	TTT	IENFD	Itch	Muscle Cramps	Warmth Influence	Cold Influence	Exercise Influence	Rest Influence	Temperature Sensation	Pain Sensation	Allodynia	Sleep Pattern
*ANO3*	(Gly1034Arg)	M	18	n	a/n	n	N	N	-	-	N	N	n	↑	Y	n
*KCNQ3*	(Val629Ile)	M	49	a/n	a/n	a/n	N	N	-	Y ↑	Y ↑	Y ↑	n	-	Y	n
*KCNQ3*	(Asp569Gly)	M	46	n	a/n	n	N	Y	-	-	-	Y ↑	-	-	Y	-
*TRPA1*	(Arg393*)	M	50	a/n	a/n	a/n	N	Y	N	N	Y ↑	-	n	-	Y	a/n
*TRPA1*	(Arg652*)	F	17	n	a/n	n	N	N	Y ↑	Y ↑	Y ↑	N	↓	↓	Y	a/n
*TRPA1*	(Met689Val)	F	34	n	a/n	a/n	Y	N	Y ↑	-	Y ↑	Y ↑	-	↑	Y	a/n
*TRPA1*	(Tyr327Cys)	M	45	n	-	a/n	N	Y	-	-	-	-	-	-	-	-
*TRPM8*	(Arg368Trp)	M	45	n	a/n	a/n	N	N	-	-	-	-	↓	n	-	-
*TRPV1*	(Arg579Cys)	M	43	n	a/n	n	Y	Y	N	N	-	Y ↑	↓	n	Y	a/n

p. position, location in protein; M, male; F, female; NCS, nerve conduction study; n, normal; a/n, abnormal; -, data incomplete; TTT, thermal threshold testing; IENFD, intraepidermal nerve fiber densities; Y, yes; N, no; ↓, decreased; ↑, increased. Patients with incomplete data not included in the table.

**Table 5:** Clinical features of SFN patients without an ICG/VGSC variant.

Feature	NCS N = 308	TTT N = 305	IENFD N = 307	Temperature Sensation N = 204	Pain Sensation N = 145	Sleep Pattern N = 130	
Normal	297, 96.4%	25, 8.2%	178, 58%	120, 58.8%	73, 50.3%	35, 26.9%	
Abnormal	11, 3.6%	280, 91.8%	129, 42%	84, 41.2% (1, 0.5% ↑, 83, 40.7% ↓)	72, 49.7% (33, 22.8% ↑, 39, 26.9% ↓)	95, 73.1%	
**Feature**	**Itch** **N = 308**	**Muscle Cramps** **N = 308**	**Warmth Influence** **N = 172**	**Cold Influence** **N = 169**	**Exercise Influence** **N = 195**	**Rest Influence** **N = 123**	**Allodynia** **N = 211**
yes	25, 8.1%	56, 18.2%	84, 48.8% (63, 36.6% complains ↑, 21, 12.2% complains ↓)	82, 48.5% (54, 32% complains ↑, 28, 16.6% complains ↓)	161, 82.6% (144,. 73.8% complains ↑, 17, 8.7% complains ↓)	54, 68.3% (45, 36.6% complains ↑, 39, 31.7% complains ↓)	193, 91.5%
no	283, 91.9%	252, 81.8%	88, 51.2%	87, 51.5%	34, 17.4%	39, 31.7%	18, 8.5%

NCS, nerve conduction study; n, normal; a/n, abnormal; -, not determined; TTT, thermal threshold testing; IENFD, intraepidermal nerve fiber densities; Y, yes; N, no; ↓, decreased; ↑, increased.

**Table 6:** Complaints reported in the Small Fiber Neuropathy Symptom Inventory Questionnaire (SFN-SIQ) by SFN patients carrying a variant in ICG.

Gene	Variant	Sweating Change	Diarrhea	Constipation	Micturition Problem	Dry Eyes	Dry Mouth	Dizziness on Standing	Palpitations	Hot Flashes	Hypersensitivity of Leg’s Skin	Burning Feet	Sheet Intolerance	Restless Leg
ANO3	(Gly1034Arg)	4	1	1	3	3	4	2	-	1	4	4	4	4
KCNQ3	(Val629Ile)	2	2	2	2	1	2	1	1	2	1	3	2	3
KCNQ3	(Asp569Gly)	3	2	2	3	2	2	2	2	2	3	4	3	4
TRPA1	(Arg393*)	-	2	3	3	1	3	2	1	2	4	4	4	4
TRPA1	(Arg652*)	3	2	3	2	1	3	3	2	2	3	3	3	3
TRPA1	(Met689Val)	3	3	3	2	4	4	3	3	3	4	3	4	4

Number value 1–4 expresses frequency of complaints; 1, never; 2, sometimes; 3, often; 4, always; -, not determined.

**Table 7:** Patients with SFN without a (potentially) pathogenic ICG/VGSC variant reporting Small Fiber Neuropathy Symptom Inventory Questionnaire (SFN-SIQ) complaints.

Frequency of Complaint	Sweating Change N = 268	Diarrhea N = 272	Constipation N = 275	Micturition Problem N = 273	Dry Eyes N = 273	Dry Mouth N = 273	Orthostatic Dizziness N = 271	Palpitations N = 268	Hot Flashes N = 270	Hypersensitivity of Leg’s Skin N = 273	Burning Feet n = 269	Sheet Intolerance N = 272	Restless Leg N = 272
never	56, 20.9%	114, 41.9%	115, 41.8%	91, 33.3%	95, 34.8%	54, 19.8%	73, 26.9%	105, 39.2%	86, 31.9%	52, 19.0%	21, 7.8%	70, 25.7%	36, 13.2%
sometimes	94, 35.1%	110, 40.4%	86, 31.3%	75, 27.5%	87, 31.9%	97, 35.5%	130, 48.0%	108, 40.3%	94, 34.8%	75, 27.5%	35, 13.0%	76, 27.9%	80, 29.4%
often	85, 31.7%	44, 16.2%	55, 20.0%	80, 29.3%	65, 23.8%	91, 33.3%	59, 21.8%	54, 20.1%	86, 31.9%	70, 25.6%	101, 37.5%	68, 25.0%	91, 33.5%
always	33, 12.3%	4, 1.5%	19, 6.9%	27, 9.9%	26, 9.5%	31, 11.4%	9, 3.3%	1, 0.4%	4, 1.5%	76, 27.8%	112, 41.6%	58, 21.3%	65, 23.9%

N, number of patients that completed the SFN-SIQ related question.

**Table 8:** Gene panel.

Ion Channel Family	Gene	OMIM Number	Full Gene Name
Anoctamins	*ANO1*	610108	Anoctamin 1, calcium activated chloride channel
	*ANO3*	610110	Anoctamin 3
Non-selective cation channels	*HCN1*	602780	Hyperpolarization activated cyclic nucleotide-gated potassium channel 1
Potassium channels	*KCNA2*	176262	Potassium voltage-gated channel, shaker-related subfamily, member 2
	*KCNA4*	176266	Potassium voltage-gated channel, shaker-related subfamily, member 4
	*KCNK18*	613655	Potassium channel, subfamily K, member 18
	*KCNN1*	602982	Potassium intermediate/small conductance calcium-activated channel, subfamily N, member 1
	*KCNQ3*	602232	Potassium voltage-gated channel, KQT-like subfamily, member 3
	*KCNQ5*	607357	Potassium voltage-gated channel, KQT-like subfamily, member 5
	*KCNS1*	602905	Potassium voltage-gated channel, delayed-rectifier, subfamily S, member 1
Transient receptors	*TRPA1*	604775	Transient receptor potential cation channel, subfamily A, member 1
	*TRPM8*	606678	Transient receptor potential cation channel, subfamily M, member 8
	*TRPV1*	602076	Transient receptor potential cation channel, subfamily V, member 1
	*TRPV3*	607066	Transient receptor potential cation channel, subfamily V, member 3
	*TRPV4*	605427	Transient receptor potential cation channel, subfamily V, member 4

## Supplementary Materials

The following supporting information can be downloaded at: https://www.mdpi.com/article/10.3390/ijms232214095/s1.
