# Supplementary material for: Peripheral Ion Channel Genes Screening in Painful Small Fiber Neuropathy
Source: Int J Mol Sci. 2022 Nov 15;23(22):14095. doi: 10.3390/ijms232214095 (PMC9696564; doi:10.3390/ijms232214095)
Supplement: Supplementary file 1 [file ijms-23-14095-s001.zip › ijms-1978410-supplementary.pdf]

Supplementary materials

Clinical characteristics of patients with Ion Channel variant

<u>Patient with *ANO3* c.2656A>T variant</u>

The patient is in their 50s presented with burning and aching pain with severe tactile allodynia at lower limbs, lateral and medial thigh at distal leg, since 3 years. The complaints are increasing during rest. Wearing clothes, tactile stimuli and cold temperature were reported as pain triggers. The mean pain at recruitment was VAS>3. The mean pain intensity was PI-NRS=5.

Patient suffered from dysautonomia: skin discoloration, distal vasoparalisis and orthostatic dizziness.

No family members reported similar complaints.

The patient has normal NCS and reduced IEND both at distal leg and proximal thigh (IEND value=2.2, IEND 5e percentile value =3.4). TTT was abnormal. Quantitative Sensory Testing (QST) at foot and at proximal thigh indicated increased warm detection thresholds, and reduced heat pain thresholds (allodynia).

Patient took pain medications: Duloxetine 60 mg (45% pain relief) and Gabapentin 2400 mg.

<u>Patient with *ANO3* c.3100G>C variant</u>

The patient is in their 60s with onset of complaints at age 18. Patient has allodynia, hypersensitivity of leg's skin, burning feet, sheet intolerance and restless leg syndrome. Patient does not report any trigger influencing pain, however rest has been reported to reduce complaints. Temperature sensation was normal, pain sensation was increased. Maximal pain was VAS=9.7.

Patient suffered from dysautonomia: dry mouth, dry eyes, sweating change, micturition problems and sometimes orthostatic dizziness.

The positive family history has been reported. Mother had painful arms and legs without underlying reason. Cousin, son of mothers' sister, also have painful arms and legs. No underlying reason was found.

The patient has normal NCS and normal IEND, IEND value=7.6, IEND 5e percentile value =2.8. Thermal Threshold Testing (TTT) was abnormal.

The patient took pain medication: amitriptylin and pregabalin, which were effective against the pain. However, with the medication, the patient is less alert (probably due to amitriptylin).

Patient with *KCNK18* c.1107del variant

The patient is in their 60s presented with progressive complaints since three years. It started with a tight feeling in both feet (at the right side more than on the left side), subsequently, developed a burning sensation and tingling in the feet, which extended to both legs, hands and arms. The complaints increased at night, during exercise and stressful events. During the day, warmth alleviated the complaints. At night, it was the opposite. Therefore, sometimes patient used icepacks at body. Some days feet discolored red.

The patient suffered from dysautonomia: hot flashes, dry eyes and mouth, constipation and increased transpiration.

No family members reported similar complaints.

Neurological examination showed absent Achilles tendon reflexes. NCS showed no large nerve fiber involvement. The diagnosis of SFN was confirmed in different center. TTT and IENFD have not been performed at our centre.

Patient took pain medication: amitriptylin, gabapentin, pregabalin, tegretol and oxycontin without any effect. Amitriptylin caused weight increase. Cannabis on medical receipt improved sleep quality.

Patient with *KCNQ3* c.1706A>G variant

The patient is in their 40s presented with a stabbing pain in the right foot since two years, which has extended to both feet, legs, hands, lower back, groins, buttocks, shoulders and cheeks. Both feet felt numb. Touching the skin was sometimes annoying. Patient experienced itch at different parts of the body. Patient reported burning feet and restless legs. The complaints were worse at rest, leading to the sleeping problems. Temperature did not influence the pain. Pain intensity was evaluated in VAS score, the maximal pain was 8.5.

Besides increased transpiration and micturition problems, patient did not report other autonomic complaints.

No family members reported similar complaints.

Neurological examination was without any abnormalities. NCS showed no signs of large nerve fiber damage. TTT showed abnormal cold and warmth sensation in both feet. The IENFD in skin biopsy was normal (5.0/mm, 5th percentile 4.4/mm).

Patient took amitriptylin 75 mg twice a day, which had a positive effect on sleep, but did not relief the pain.

Patient with *KCNQ3* c.1885G>A variant

The patient is in their 60s presented with an annoying feeling in both feet since 15 years. Gradually this feeling had changed to a numb sensation. The right foot felt continuously tight, with alternating burning, cold and painful tingling sensations. Sometimes the complaints extended to the knees. In addition, the patient experienced tingling in both hands, but those were well tolerated. Exercise and cold temperatures increased the complaints, while warmth decreases the pain. Also during the day, the complaints worsened at rest. Pain intensity was evaluated in VAS score, the maximal pain was 8.7.

The patient suffered from dysautonomia: sometimes orthostatic dizziness and impotence.

NCS showed abnormalities compatible with a severe axonal sensorimotor peripheral neuropathy. The temperature levels for warmth and cold were disturbed at both feet. Skin

biopsy showed a decreased IENFD of 0.7/mm (5th percentile 2.8/mm). Neurological examination showed an abnormal sensation (vibration sense, pain sensation and propriocepsis) at the toes and an areflexia of all tendon reflexes.

Additional blood tests revealed a positive antinuclear antibodies (ANA), elevated gamma-GT and triglycerides, and a monoclonal gammopathy IgG type Lambda.

Patient took pregabalin, which was effective for pain treatment.

Patient with *TRPA1* c.932C>A variant

The patient is in their 60s. Patient was diagnosed with SFN, and suffered from Ehlers-Danlos syndrome and neuropathic pain. The IENFD 10 cm above the ankle was decreased.

Patient with *TRPA1* c.980A>G variant

The patient is in their 50s presented with a numb sensation in both legs since four years. Three years after this presentation patient developed painful cramps in fingers, lower legs and feet. Exercise worsened the cramps. Pain intensity was evaluated in VAS score, the maximal pain was 10.

The patient suffered from dysautonomia: dry mouth and dizziness (probably side effect of medication).

Family history showed no persons with similar complaints.

Neurological examination showed a hypoesthesia at both feet and ankles. Repetitive NCS showed no signs of large nerve fiber involvement. The IENFD 10 cm above the ankle was decreased: 2.1/mm (5th percentile 4.0/mm). TTT was not performed.

Patient took pain medication: gabapentin gave too much side effects (300 mg in the morning and 100 mg in the evening), duloxetine 30 mg twice a day did not have effect, amitripylin 100

mg a day did not relief the complaints, pregabalin 150 mg twice day caused dizziness, tramadol and methadone gave too much side effects.

Patient with *TRPA1* c.1177C>T variant

The patient is in their 50s presented with a disturbed sensation, tingling and pain in the right foot that has extended to the knee and left leg. The complaints has started eight years ago after a nerve root block. The pain was continuously present, but differed in intensity. Exercise increased the pain. During the weekend the pain decreased. The sleeping pattern was disturbed by the pain. The patient could stand the blankets on feet, but touching the mattress was painful. The maximal pain score was VAS=10.

Patient suffered from dysautonomia: dry eyes, hyperhidrosis, alternating diarrhea and constipation, micturition problems, orthostatic dizziness.

No family members with similar complaints.

Neurological examination was normal. NCS showed signs of a mild axonal sensorimotor peripheral neuropathy (decreased CMAP amplitude peroneal and tibial nerve, decreased conduction velocity sural nerve, prolonged F-response peroneal nerve). TTT showed abnormal levels for warmth and cold sensation in both feet. The IENFD 10 cm above the ankle was decreased: 3.2/mm (5th percentile 3.5/mm).

Patient took pain medication: pregabalin (caused side effects), amitripylin (no effect on the pain), duloxetin (stopped because of hypertension, no effect on the pain, also side effects of impotence and obstipation).

Patient with *TRPA1* c.1954C>*T* variant

The patient is in their 20s presented with tingling, numbness and sometimes a burning sensation in both hands (right hand most affected) and lower legs (left leg most affected) since three years. Exercise and temperature changes increased the complaints. Rest alleviated the

sensations. Blankets and shoes were painful at the skin. The night rest was disturbed by an annoying feeling in the legs. Pain intensity was evaluated in VAS score, the maximal pain was 10.

Patient reported several autonomic complaints: dry mouth, hyperhidrosis, facial flushing, constipation, orthostatic dizziness, gastroparesis and diminished lubrication.

Daughter of father has same complaints and was diagnosed with Multiple Sclerosis.

Neurological examination was normal. NCS showed no signs of large nerve fiber involvement. TTT showed only a disturbed level for warmth sensation at the right foot. Skin biopsy showed a normal IENFD of 12.0/mm (5th percentile 8.4/mm). Additional blood tests showed an elevated Sol-Il 2 receptor value.

Patient took diclofenac that did not have effect.

Patient with *TRPA1* c.2065A>G variant

The patient is in their 30s presented with a burning sensation, tingling and a numbness feeling in both hands and feet since two years. Besides, the patient experienced itch in arms, lower legs and neck. The symptoms were continuously present, but most intense during rest. Exercise and a warm shower increased the pain. A cold shower alleviated the complaints. During coldness, hands become white. Shoes and blankets were painful to the skin. The night rest was disturbed by the pain. The maximal pain was VAS=8.6.

Patient suffered from dysautonomia: dry eyes and mouth, hyperhidrosis, hot flashes, cardiac palpitations, orthostatic dizziness, swallowing difficulties, micturation problems and alternation diarrhea and constipation.

The mother reported palpitations and burning sensations in hands and feet.

Neurological examination and NCS were normal. TTT showed abnormal levels for cold sensation at both feet. The IENFD 10cm above the ankle was decreased: 6.5/mm (5th percentile 7.1/mm).

Additional blood tests showed ANA and decreased vitamin B12.

Patient took pregabalin and tramadol, but the effect is unclear.

Patient with *TRPA1* c.3136A>G variant

The patient is in their 40s . In 2003 patient started to complain of pain in the left knee that could not be connected with an orthopedic diagnosis. The pain was described as burning and eventually spread to left calf and later to the foot. The pain increased with movement. The mean pain at recruitment was VAS>3.

Autonomic complaints not reported.

The patient is one of two siblings with *TRPA1* c.932C>A variant and burning pain worsening with movement and responding to neuropathic pain medications.

The patient has normal NCS and reduced IEND (IEND value=3.6, IEND 5e percentile value =4.4).

The patient took pain medication: Tolep 300, Lioresal 25 mg, Neurontin 100 mg, Lyrica 150, all poorly tolerated. Good response with Laroxyll, but urinary retention appears, later developed intolerance to Lyrica (tachycardia and general malaise), Xeristar suspended for ineffectiveness.

Second patient with *TRPA1* c.3136A>G variant

The patient is in their 40s presented with burning in the thighs with progressive worsening (acute onset in May 2012). The mean pain at recruitment was VAS>3. Severe pain causing awakening was reported during night and increased activity was recognized as pain provoking factor.

No autonomic complaints were reported.

One of the sibling of the patient has similar complaints (included in this study, also carrying *TRPA1* c.3136A>G).

The patient carried out in Motor Evoked Potential (MEP), Somatosensory Evoked Potentials (SEPs), Electroneuronography (ENoG), Electromyography (EMG) which resulted in the norm (2012), venous doppler in the norm. The IENFD was decreased.

The patients is ongoing hypothyroidism therapy. Lumbar discopathies (from L2 to L5 bulging discs without impingement, in L5-S1 small not severe hernia).

The patients was undergoing steroid therapy with Muscoril with good response, but quick reoccurrence of symptoms appeared after suspension. Patient took pain medication: Lyrica 25 mg, lexotan 10 drops x 3, zoloft 25 drops were recommended under psychiatric counseling, with a moderate benefit. The patient suspended zoloft on their own.

<u>Patient with *TRPM8* c.665A>G variant</u>

The patient is in their 50s presented with complaints of sharp and stabbing pain, numbness at feet and distal legs, since 8 years. Pain was increased at night and due to movement and hot temperature.

Patient suffered from dysautonomia: distal anhidrosis, orthostatic hypotension, signs of peripheral vasoparalysis, constipation.

No family members with similar complaints.

The patient has normal NCS and reduced IEND at distal leg (IEND value=1.1/mm), not at proximal thigh. Quantitative Sensory Testing (QST) displayed high warm and cold detection thresholds, and reduced heat pain thresholds (hyperalgesia).

The patient took pain medication: gabapentin 2400 mg + amytriptiline 25 mg with mild pain improvement (30%).

<u>Patient with *TRPM8* c.1102C>T variant</u>

The patient is in their 50s presented with a burning sensation at the dorsal side of the left upper leg since one year. Besides, the patient experienced burning pain alternating in both legs, knees, calves and feet, every evening both feet were burning. The patient could not stand the blankets at the feet. The patient suffered from diabetes type II and had a history of alcohol abuse.

The patient reported various autonomic complaints including dry mouth, hyperhidrosis and constipation.

Neurological examination showed allodynia at the dorsal side of the left leg. NCS were normal. TTT showed disturbed warmth sensation at both hands and an abnormal cold sensation at the left hand. Skin biopsy showed a decreased IENFD of 2.6/mm (5th percentile 3.5/mm).

Patient took medication: pregabalin 75 mg twice a day, but after one week stopped because of dizziness. It is unclear whether it influenced the pain intensity.

Patient with *TRPM8* c.2945C>T variant

The patient is in their 30s presented with pelvic pain (stabbing and electric shock-like pain and itch) and feet dorsum. The symptoms started at 19 years old and worsened in the last 4 years. Patient was diagnosed with SFN, without known underlying case.

Patient with *TRPV1* c.914T>G variant

The patient is in their 40s and was diagnosed with SFN, without known underlying case. Patient complained for burning mouth, since 2 years. The mean pain at recruitment was VAS>3. The mean pain intensity was PI-NRS=6.

The patient suffered from dysautonomia: dry mouth and restless leg syndrome.

The IENFD 10 cm above the ankle was decreased

The patient took Gabapentin, which was suspended due to intolerance (gastrointestinal disturbances and vertigo).

Patient with *TRPV1* c.1348A>G variant

The patient is in their 40s presented with pain in the hands from the beginning of 2014 in the right hand and after three months, also in the left hand. Pain was prevalent at rest and affected predominantly the intrinsic musculature of the hand (palm), not the joints. No difficulty in opening the fingers after clench fists. Since 2015 the pain has spread to all fingers, it gets worse in position firm, slight improvement if moving fingers. Pain is worsening at rest. No pain changes in warm or cold temperature was observed. The mean pain at recruitment was VAS>3. Usually the pain oscillates between VAS=3-4, when worsened it reached VAS=7-8.

Performed ENoG indicated damage at C6C7 bilateral. SEP laser, Nuclear Magnetic Resonance (NMR), Laser-evoked potentials (LEPs) were normal. Patient has reduced IENFD, IEND value=3.0, IEND 5e percentile value =4.4.

Patient took pain medications: Lyrica 25 mg, withdrawn due to excess sleep.

Patient with *TRPV1* c.1735C>T variant

The patient is in their 50s presented with restless legs and tingling in both lower legs since six years. After a tibia fracture operation in 2009 the patient experienced an increase of symptoms. The lower legs felt like they were tangled with stretch bandage. During rest, tingling started in both legs. The patient experienced continuously a pins and needles sensation in the hands, arms and neck. The complaints increased at night interfering with night rest. Temperature did not influence the tingling or pain. After a warm shower the patient experienced severe itch at lower legs. The temperature sensation was decreased. Blankets, shoes and socks were painful to the skin. Pain intensity was evaluated in VAS score, the maximal pain was 6.
Patient reported the following autonomic symptoms: hyperhidrosis and urine incontinence.

Family history revealed no persons with similar complaints.

Neurological examination and NCS were normal. TTT showed abnormal levels for warmth and cold sensation in both feet. The IENFD in skin biopsy was normal: 7.6/mm (5th percentile 4.4/mm).

Patient took oxycontin 5 mg, which reliefs the pain at night.

Patient with *TRPV3* c.1242+1G>A variant

The patient is in their 80s presented with complaints of swollen legs sensation followed by intense burning, heat and sensation of constriction. At the same time, the patient had hypertensive episodes with facial flushing, associated with a sensation of intense heat with fecal sphincter urgency. The patient has tactile allodynia.

The patient has normal NCS and reduced IENFD, IENFD value=1.2, IENFD 5e percentile value=1.7.

The patent took pain medications: Lyrica, which was suspended and substituted with duloxetine (no data on drug response).

Patient with *TRPV3* c.2006T>C variant

The patient is in their 50s presented with intra-auricular itch and pain and burning in the feet soles, diffusing till the legs. The complaints stared in 2010 and subsequently pain symptoms were spreading in the arms and trunk. Patient reported episodes of itching in the cervical region, in the auricles, in the head and in the genital area.

No family members with similar phenotype.

The patient has normal NCS and reduced IENFD, IENFD value=2.4.

The patient has hypothyroidism since 2009, vitamin D deficiency and osteopenia in therapy with cholecalciferol.

The patent took pain medication: duloxetine with partial benefit in the beginning, later it was discontinued due to ineffectiveness. Lyrica was ineffective. The patients is taking Palexia 150 mg x 2 with partial benefit, Diazepam 5-6 drops in the evening, with benefit on sleep and Atarax in the evening.
